# Seasonal variation in air pollutant levels and its effects on the sex ratio at birth on Fukue island, Japan

**DOI:** 10.1186/s12889-023-17418-5

**Published:** 2023-12-11

**Authors:** Hiroaki Arima

**Affiliations:** Department of International Health and Medical Anthropology, Institute of Tropical Medicine, Nagasaki University, 1-12-4 Sakamoto, Nagasaki City, Nagasaki 852-8523 Japan

**Keywords:** Air Pollutant, Oxidant, Sex ratio at birth, Island, Japan

## Abstract

**Background:**

In general, a slightly higher number of boys are born than girls, and the sex ratio at birth (number of male births/number of female births) is reported to be 1.03–1.07 in many countries. However, pregnant women exposed to high levels of atmospheric particulate matter have a reduced sex ratio at birth. Exposure to air pollutants can also lead to premature birth, suggesting that inflammation within the body may affect pregnancy maintenance and fetal development. On the other hand, the effects of air pollutants carried from afar by monsoons on the sex ratio at birth in downstream areas have not been evaluated. We focused on the Goto Islands, where various air pollutants flow from the Eurasian continent. The objective of this study was to clarify the effects of the atmospheric level of each pollutant on the sex ratio at birth on the Goto Islands.

**Methods:**

We extracted observation data of particulate matter 2.5, sulfur dioxide, oxidants, nonmethane hydrocarbons, and methane from the National Institute for Environmental Studies database. In addition, the monthly sex ratio at birth was calculated using birth data from the National Statistics Center. To evaluate the effect of substance exposure just before fertilization on the sex ratio at birth, we analyzed the relationship between the observed pollutant level and the sex ratio at birth 9 months later. A stepwise generalized linear model was used to analyze the effects of air pollutant levels on the sex ratio at birth.

**Results:**

The observed values for all pollutants were significantly different between seasons, including the particulate matter 2.5 (*p* < 0.0001), sulfur dioxide (*p* = 0.0026), oxidant (*p* < 0.0001), nonmethane hydrocarbon (*p* < 0.0001), and methane (*p* < 0.0001) values. In the target population in the target period, the total number of births was 1835, and the sex ratio at birth was 0.967. Univariate analysis showed that the values of particulate matter 2.5 (*p* = 0.0157) and oxidants (*p* = 0.0047) correlated negatively with the sex ratio at birth. In addition, the results of multivariate analysis using the stepwise method in the model equation indicated that every 1 ppm increase in the observed OX value resulted in a 0.311 decrease in the sex ratio at birth (*p* = 0.0034).

**Conclusions:**

We evaluated the relationship between seasonal variations in air pollutant levels and the sex ratio at birth 9 months later on the Goto Islands. We found that an increase in oxidant levels just before and after conception may be a risk factor for a lower sex ratio at birth. Due to the previously reported vulnerability of male fetuses, females who become pregnant when air pollutant concentrations are high may be more likely to have a female baby. It is necessary to evaluate the effects of oxidants on various aspects of pregnancy and childbirth.

**Supplementary Information:**

The online version contains supplementary material available at 10.1186/s12889-023-17418-5.

## Background

In humans, the theoretical sex ratio at fertilization is 1:1, but the miscarriage rate for female fetuses is usually slightly higher than that for male fetuses [1]. Therefore, in reality, the human sex ratio (number of male births/number of female births) exceeds 1 and is reported to be 1.03–1.07 in many countries [2].

There are reports that the sex ratio at birth is reduced when mothers or fathers are exposed to chemicals. For example, children born to pregnant women exposed to tobacco [3], polychlorinated biphenyls (PCBs) [4], and methylmercury [5] have a reduced sex ratio at birth. A reduction in the sex ratio at birth is also observed when fathers are exposed to dioxin [6]. Focusing on air pollutants, in Brazil, it has been reported that the sex ratio at birth decreased in pregnant women when the concentration of particulate matter 2.5 (PM2.5) was high [7]. PM2.5 causes inflammation in the lungs and increases production of cytokines [8]. Maternal inflammation is caused by PM10 and NO_2_ exposure due to environmental pollution [9]. It has also been found that increased exposure to particulate matter 10 (PM10) and PM2.5 in men decreases the Y/X chromosome ratio in sperm [10]. Furthermore, it has been revealed that psychological and physical stress that induce inflammation reduce the sex ratio at birth [11]. Maternal psychological and physical stress is associated with increased inflammation, and male fetuses are more vulnerable to environmental changes than female fetuses [12]. Furthermore, since exposure to various substances is known to be a risk factor for preterm birth [13, 14], exposure to substances may affect various aspects of pregnancy through inflammation and be involved in a decrease in the sex ratio at birth. This study explored the possibility that various air pollutants affect the sex ratio at birth.

**Fig. 1:**
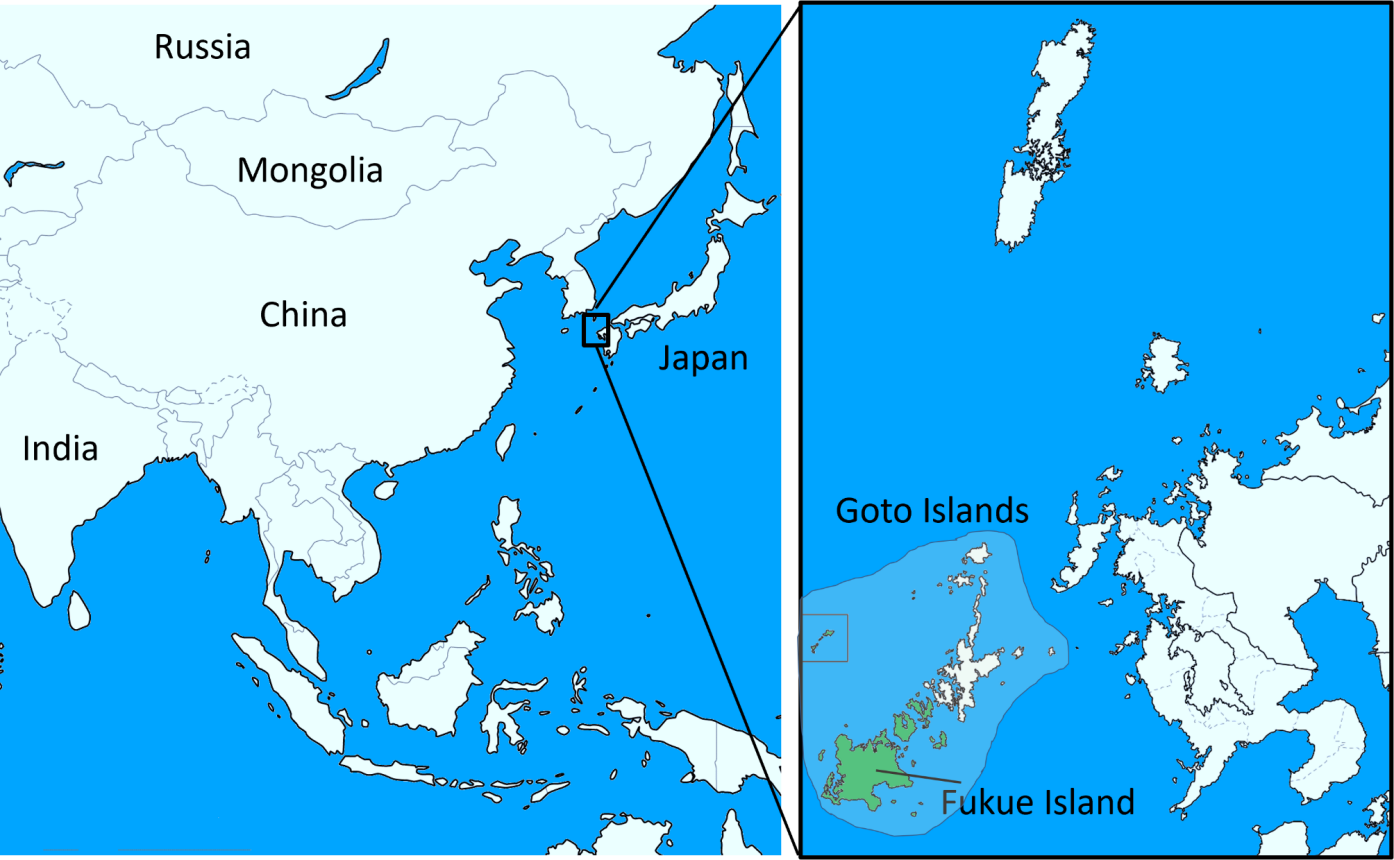
Locations of the Goto Islands and Fukue Island. The Goto Islands are divided into two cities. In this study, we analyzed the sex ratio at birth in the Goto Islands (the green islands), which include Fukue Island, where air pollutant levels were measured

Fukue Island is located in the Goto Islands of Nagasaki Prefecture, Japan (Fig. 1). Aerosol mass spectrometry conducted on Fukue Island has revealed that air pollutants from East Asia, especially China, are carried by air currents and reach the island [15–18]. In these studies, the movement of airflow over a 48-hour period was analyzed, and it was found that pollutants from northern China passed over South Korea and reached Fukue Island. It has been reported that PM2.5 and SO_2_ levels on Fukue Island actually change depending on the wind direction and speed. According to the Japan Meteorological Agency, westerly winds flow from southern China to Japan (parallel to the equator) throughout the year [19]. During the winter period from December to February, a high atmospheric pressure system forms near Siberia, and a low atmospheric pressure system develops near the Aleutians, causing monsoon winds to blow from northern China toward Japan [15]. In addition, the Environmental Observatory located on Fukue Island, which is a passageway for air currents, was developed by Japan’s National Institute for Environmental Studies to measure pollutants brought to Japan from overseas, not those generated within Japan. For these reasons, this study focused on Fukue Island to evaluate the effects of airborne pollutants on the sex ratio at birth. To date, no investigation on how the concentration of atmospheric substances affected by air currents impacts sex determination during fertilization and after implantation in areas downstream of seasonal winds has been conducted. The objective of this study was to clarify the seasonal variations in air pollutant levels observed on Fukue Island and the effects of atmospheric pollutant levels on the sex ratio at birth 9 months later in the Goto Islands.

## Methods

### Data collection

The Environmental Observatory of the National Institute for Environmental Studies monitors the concentrations of air pollutants in various parts of Japan and publishes the data. In this study, observational data for Fukue Island included data on PM2.5 as fine particulate matter, sulfur dioxide (SO_2_) as sulfur oxide, oxidants (OXs) as oxidizing substances, nonmethane hydrocarbons (NMHCs), and methane (CH_4_) as other hydrocarbons, which were extracted from monthly Continuous Monitoring of Air Pollution reports [20]. For all pollutants, the monthly averages of observational data were used for analysis. However, OXs are observed during the daytime (5:00 a.m. to 8:00 p.m.) since the production of OXs is affected by sunlight, and the values for each hour are aggregated. In this study, the monthly average value was used for analysis. In addition, since observations for NMHCs and CH_4_ were conducted in the morning (6:00 a.m. to 9:00 a.m.), we used the monthly average value data.

In addition, the number of births by sex in the jurisdiction of the Goto Public Health Center, published by e-Stat and managed by the Statistics Center, was extracted per month, and the monthly sex ratio (number of male births/number of female births) was calculated.

Observational data of air pollutants for 108 months (from April 2013 when observations of PM2.5 levels began in Fukue to March 2022) were used for analysis. In addition, to evaluate the effects of exposure to substances just before or after fertilization on the birth sex ratio, data on the number of births from January 2014 to December 2022 (9 months after the substance level observation period) were used.

### Statistical analysis

In accordance with the standards of the Japan Meteorological Agency, March to May is considered spring, June to August is considered summer, September to November is considered autumn, and December to February is considered winter. Air pollutant concentrations were plotted for each season, and comparisons between groups were performed using analysis of variance (ANOVA) or the Kruskal‒Wallis test. After these analyses, we used the Tukey‒Kramer test or the Steel-Dwass test as a multiple test to specifically analyze which seasons showed differences. A test method for comparison was selected based on the results of the Shapiro‒Wilk test. R studio (ver. 2022.07.2 + 576) was used for statistical analyses, and a *p* value < 0.05 indicated significance.

Statistical analysis was conducted based on the correlation coefficient of the maximal information coefficient (MIC) [21], Spearman’s correlation coefficient, linear regression analysis and a generalized linear model. Multivariate analysis was performed using a generalized linear model, including each pollutant level as an explanatory variable and the sex ratio at birth as an objective variable. Taking seasonal fluctuations into account and avoiding arbitrary variable selection, we performed the analysis using a stepwise method. As a result, only OX levels remained as an explanatory variable using the stepwise method.

## Results

### Seasonal changes in air pollutant levels

Additional Fig. 1 shows the level of each substance by observation month. Atmospheric levels of PM2.5 tended to decrease from summer to autumn and increase from winter to spring. SO_2_ and CH_4_ levels tended to decrease from summer to autumn. Conversely, the observed values for NMHCs tended to be higher in summer. On the other hand, OXs had the highest correlation coefficients with the observation months (r = 0.6121) and tended to increase, with peaks in spring and autumn.

**Fig. 2:**
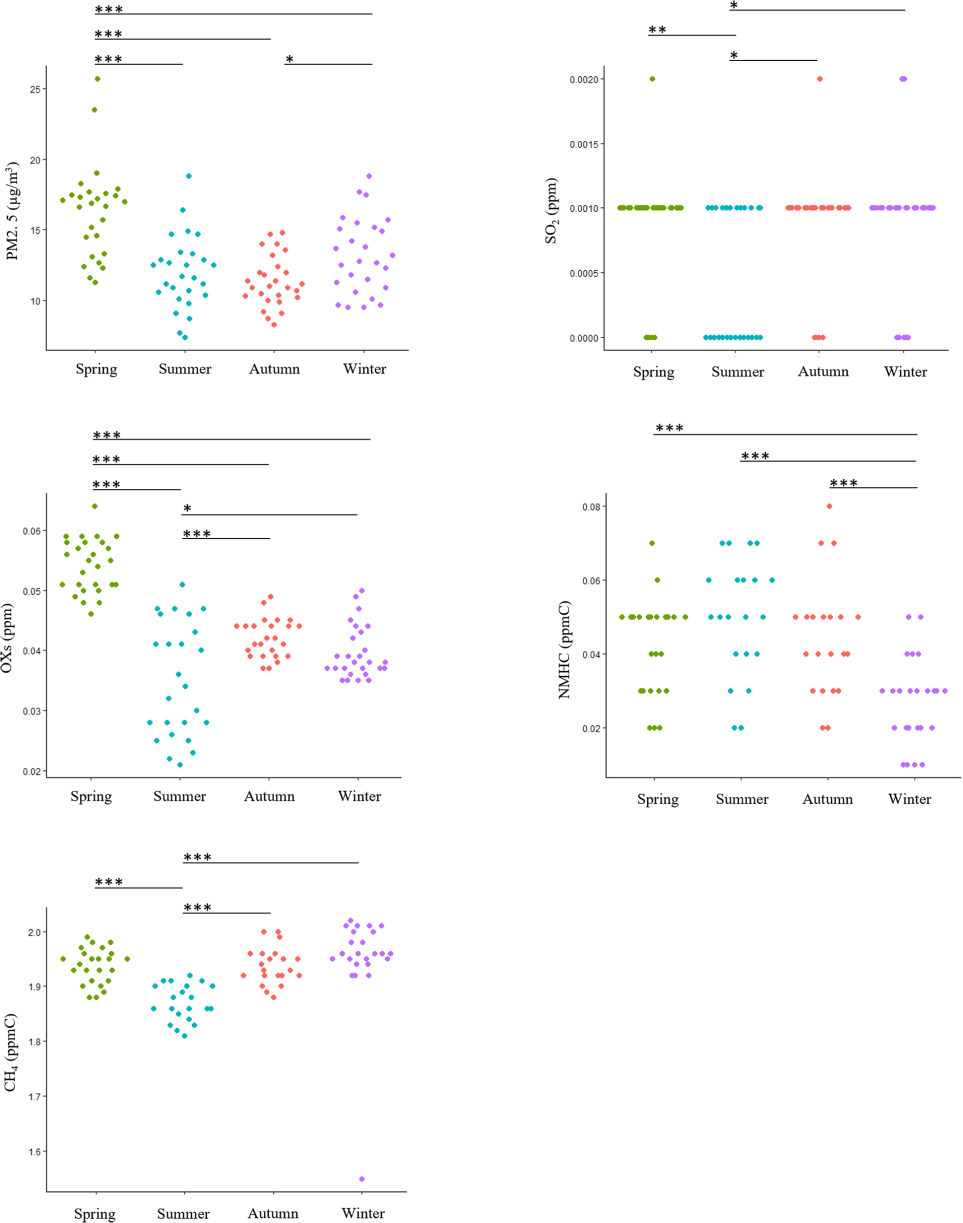
Observational data of air pollutants by season and their comparison. The normality of the observed data for each pollutant was confirmed using the Shapiro‒Wilk test. Data with a normal distribution were compared by season using ANOVA, and data without a normal distribution were compared by season using the Kruskal‒Wallis test (Additional Table 1). For air pollutants for which significance was confirmed in between-group comparisons, multiple comparisons were performed using the Tukey‒Kramer test or Steel-Dwass test, and the p values are shown in this figure. *: *p* < 0.05, **: *p* < 0.01, ***: *p* < 0.001

The observed values for each season are summarized in Table 1, and the results of seasonal comparisons of observed values are shown in Fig. 2, Additional Table 1, and Additional Table 2. PM2.5 levels were highest in spring, with significant differences observed in the other seasons (summer (*p* < 0.0001), autumn (*p* < 0.0001), and winter (*p* = 0.009)). The observed value was lowest in autumn, and a significant difference was also observed between autumn and winter (*p* = 0.0389). SO_2_ levels were lowest in summer, and significant differences were observed in the other seasons (spring (*p* = 0.0079), autumn (*p* = 0.0129), and winter (*p* = 0.0192)). For OXs, the observed values tended to be highest in spring, and significant differences were observed in the other seasons (summer (*p* < 0.0001), autumn (*p* < 0.0001), and winter (*p* < 0.0001)). In addition, the observed values of OXs were the lowest in summer, and a significant difference was observed between autumn (*p* = 0.0003) and winter (*p* = 0.0299). The observed value of NMHCs tended to be lowest in winter, and there was a significant difference from the other seasons (spring (*p* = 0.0002), summer (*p* < 0.0001), and autumn (*p* = 0.0002)). Similar to the SO_2_ value, the observed value of CH_4_ was lowest in summer, and significant differences were observed in the other seasons (spring (*p* < 0.0001), autumn (*p* < 0.0001), and winter (*p* < 0.0001)).

**Table 1:** Summary of each air pollutant level and the sex ratio at birth by season

		All			Spring			Summer			Autumn			Winter		
		Median	Q1	Q3	Median	Q1	Q3	Median	Q1	Q3	Median	Q1	Q3	Median	Q1	Q3
Air pollutant	PM2.5	12.600	10.900	15.120	16.900	13.900	17.550	11.700	10.500	13.100	11.000	10.250	12.200	12.800	11.100	15.150
	SO_2_	0.001	0.000	0.001	0.001	0.001	0.001	0.000	0.000	0.001	0.001	0.001	0.001	0.001	0.001	0.001
	OXs	0.043	0.037	0.049	0.055	0.051	0.058	0.034	0.028	0.043	0.042	0.039	0.044	0.038	0.037	0.043
	NMHCs	0.040	0.030	0.050	0.050	0.030	0.050	0.050	0.040	0.060	0.040	0.030	0.050	0.030	0.020	0.030
	CH_4_	1.930	1.900	1.960	1.945	1.910	1.960	1.860	1.850	1.900	1.930	1.920	1.960	1.960	1.948	2.000
Sex ratio at birth		0.917	0.667	1.292	1.200	0.692	1.800	1.200	0.808	1.432	0.833	0.652	1.139	0.800	0.547	1.000

Each outline of the pollutant level and sex ratio at birth are expressed as the median, 1st quartile (Q1) and 3rd quartile (Q3). The units of each pollutant are as follows: PM2.5: µg/m^3^; SO_2_: ppm; OXs: ppm; NMHC: ppmC; and CH_4_: ppmC

### Seasonal changes in the sex ratio at birth

**Fig. 3:**
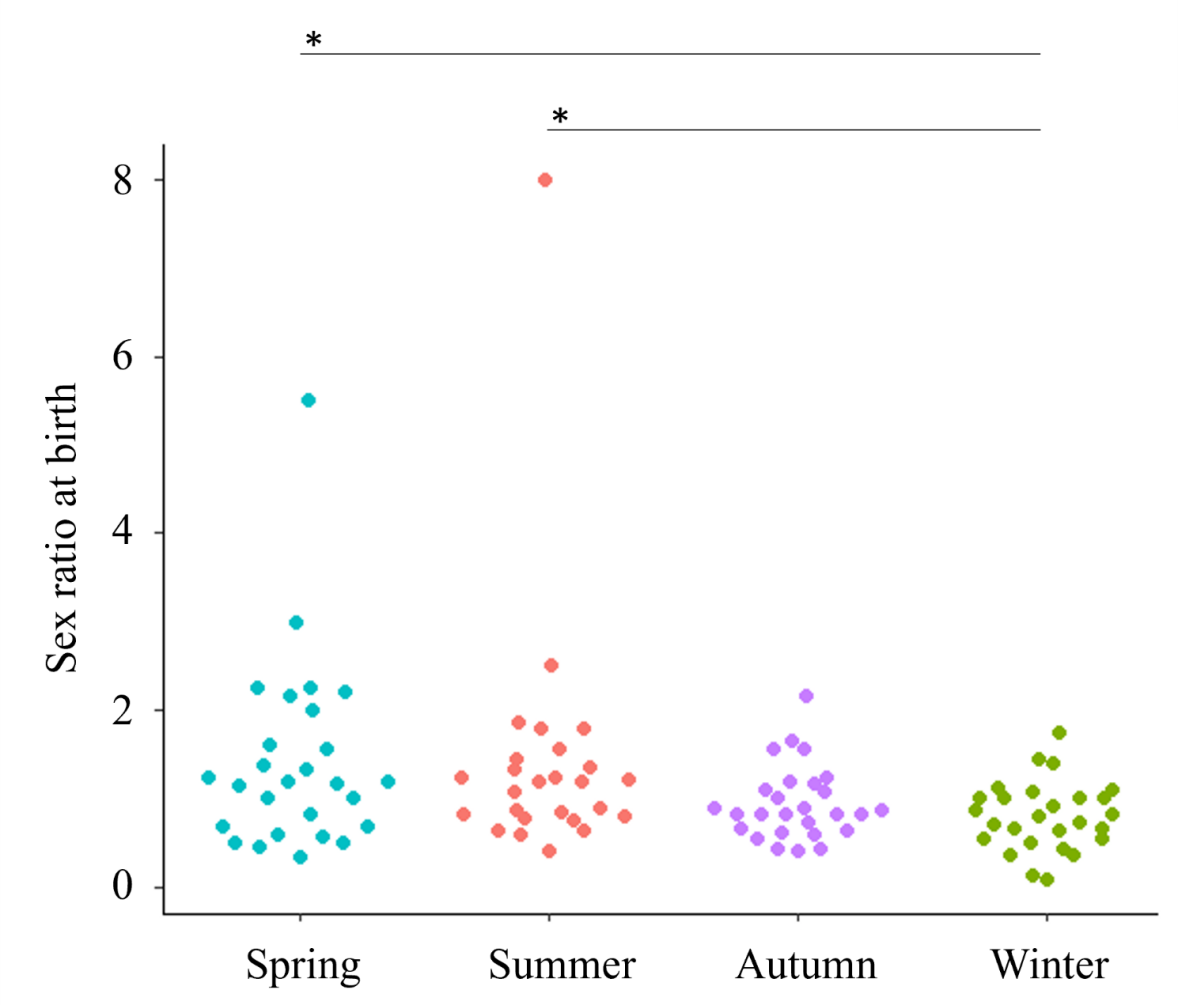
Observational data of the sex ratio at birth by season and their comparison. Normality in the sex ratio at birth data was confirmed using the Shapiro-Wilk test. The sex ratio at birth was compared between seasons using the Kruskal‒Wallis test, as there was no normality (Additional Table 1). Multiple comparisons were performed using the Steel-Dwass test, and * signs are shown in this figure. *: *p* < 0.05, **: *p* < 0.01, ***: *p* < 0.001

The total number of births on the Goto Islands during the target period was 1835, with 902 males and 933 females, and the sex ratio at birth for all term pregnancies was 0.967. When the sex ratio at birth was plotted by month, it tended to decrease slightly in winter, and the correlation coefficient of the MIC was 0.1967 (Additional Fig. 2). When classifying the sex ratio at birth by season, there were significant differences between spring and winter (*p* = 0.0303) and between summer and winter (*p* = 0.0154); the sex ratio at birth tended to be lower in winter (Fig. 3 and Additional Table 2).

### Relationship between air pollutant levels and the sex ratio at birth

**Fig. 4:**
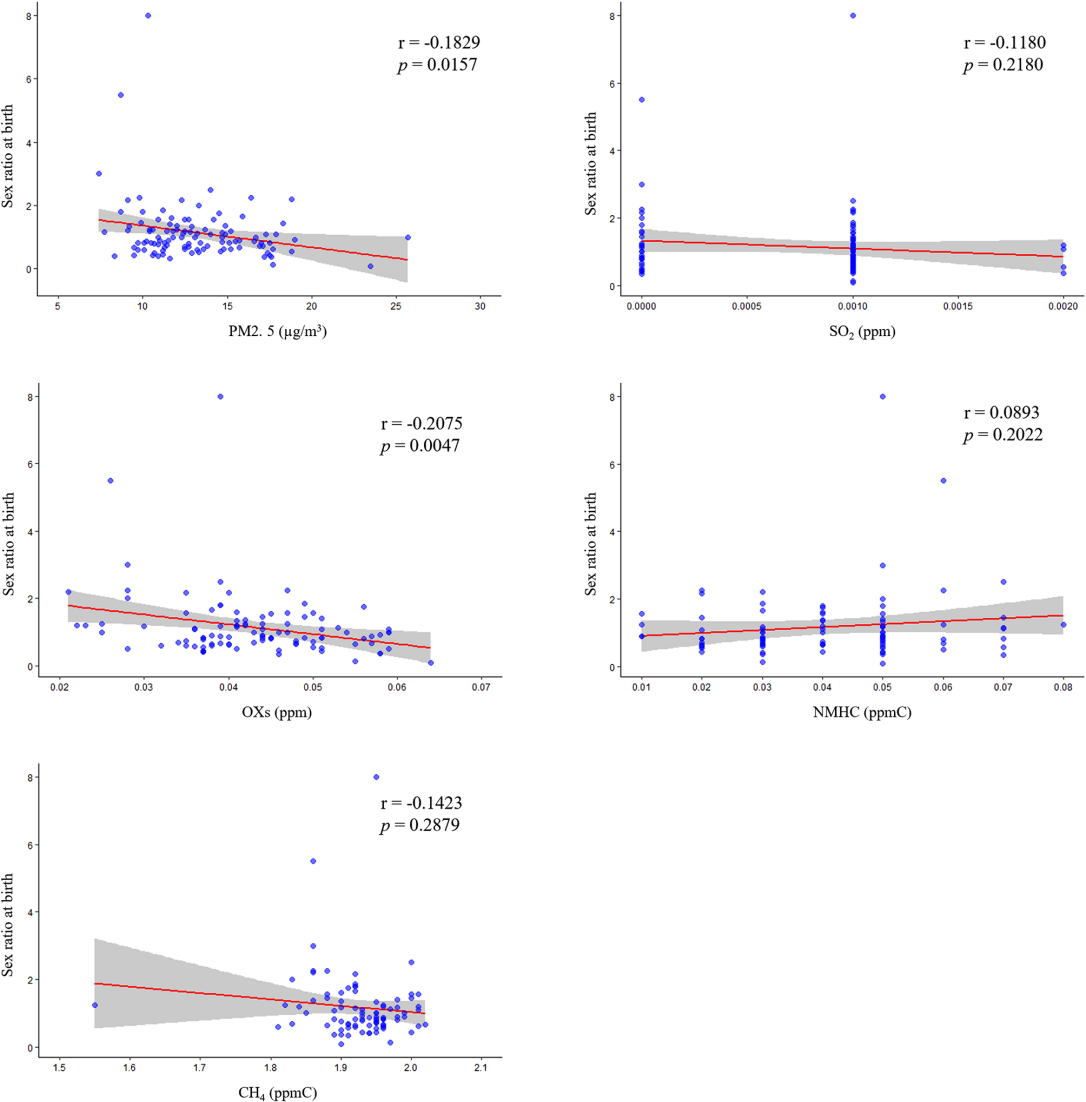
Relationship between each pollutant level and the sex ratio at birth 9 months later. The figure shows a fitted curve ± SD and the correlation coefficient of the MIC. In addition, the results of the linear regression analysis are shown as *p* values

The relationship between the observed air pollutant level and the sex ratio at birth 9 months later was plotted, Spearman’s correlation coefficient was calculated, and linear regression analysis was performed (Fig. 4, Additional Table 3). The observed levels of PM2.5 (r = -0.1829, *p* = 0.0157) and OXs (r = -0.2075, *p* = 0.0047) correlated negatively with the sex ratio at birth. The correlation coefficients between the observed level of SO_2_ (r = -0.1180), NMHCs (r = 0.0893) or CH_4_ (r = -0.1423) and the sex ratio at birth were low, and the fitted lines did not have a significant slope, even in the linear regression analysis (*p* = 0.2180, 0.2022, and 0.2879, respectively).

Additional Table 4 shows the correlations between the pollutants. A correlation was found between PM2.5 and SO_2_ (r = 0.5316) and between PM2.5 and OXs (r = 0.5092), and Fig. 2 shows that these three substances showed similar seasonal fluctuations. Analysis of the association between air pollutant levels and the sex ratio at birth using a stepwise generalized linear model revealed that OX levels were significantly associated with the sex ratio at birth (*p* = 0.0034), and the higher the observed OX value was, the lower the sex ratio at birth 9 months later (Table 2).

**Table 2:** Exploring factors associated with the sex ratio at birth by multivariate analysis

Variables	Estimate	Std.Error	95% CI	*p* value
Intercept	-3.678E-17	0.103	[-0.204 ,0.204]	1.0000
OXs	-0.311	0.103	[-0.516 ,-0.106 ]	0.0034

The results of the multivariate analysis using a generalized linear model with a stepwise method are shown. The formula for estimating the sex ratio at birth according to this analysis, which indicated that the higher the OX level was, the lower the predicted value of the sex ratio at birth, was as follows: Estimated sex ratio at birth = -0.311*OX value-3.678E-17 (constant). AIC = -5.86

## Discussion

### Seasonal variability of atmospheric concentrations

In the Japanese archipelago, including Fukue Island, air pollutants and the substances that cause them flow from the Eurasian continent due to seasonal winds. Data collected in China from June 2014 to February 2019 show that PM2.5 concentration tended to be higher in winter [22]. On Fukue Island, the target area of this study, the PM2.5 concentration tended to increase from winter to spring. It is possible that the observed value of PM2.5 tends to increase because the pollutant is blown from the Eurasian continent to the Japanese archipelago by the winter monsoon [23, 24]. On Fukue Island, OX concentrations tended to decrease from spring to winter. The main component of oxidants is ozone, which is produced in the troposphere by the reaction of nitrogen oxide (NOx) and volatile organic compounds (VOCs) with sunlight. Road transportation is the most common anthropogenic source of NOx, followed by power plants and industries. Natural sources include soil and lightning. Anthropogenic sources of VOCs include incomplete combustion of fossil fuels, the use of solvents, industries, agriculture, and biomass fuels. On the other hand, natural sources of VOCs are thought to outweigh anthropogenic sources globally [25]. A previous study reported that NOx and VOCs, or the oxidants produced by them, are carried by westerlies from Korea and China and that the observed values in Japan are high in spring [26]. In addition, studies in Japan and Hong Kong have reported that atmospheric oxidant levels peak in spring and autumn and decrease in summer due to monsoons and wind directions [27, 28]. A similar seasonal change was observed in the present study. A previous study in Japan suggested that NMHC levels tend to be highest in winter and lowest in summer due to anthropogenic emissions from the Asian continent [29]. Regarding NMHC levels, this study also observed the reverse tendency. SO_2_ data observed at the summit of Mt. Fuji in Japan showed seasonality, with an increase from winter to spring. SO_2_ fluctuates with nitrogen oxide and 222Rn, but it is not clear whether this is truly continental [30]. Therefore, the seasonal changes in the air pollutants targeted in this study are generally similar to those in previous studies.

### Relationship between air pollutants and pregnancy and childbirth

If different types of substances flow in the same air current at the same time, it is possible that the observed values of different substances on Fukue Island may correlate. However, since we wanted to observe the effects of each substance on the sex ratio at birth, we chose all these variables because it is possible that even if some correlation was observed, it would be necessary to incorporate all substances into the multivariate analysis. The results of this study showed that the sex ratio at birth tended to decrease in pregnant women who had exposure to high levels of oxidants in the air during the period of conception.

It has been reported that exposure to ozone, the main oxidant, during early pregnancy can lead to shortened gestation and increased preterm birth rates [31, 32]. To date, various studies have suggested that maternal smoking [33, 34], PM2.5 exposure [7, 13], and psychological stress exposure [11, 35] are risk factors for preterm birth and reduce the sex ratio at birth. As a phenomenon commonly occurring in pregnant women, the promotion of inflammatory reactions is mentioned. For example, smoking increases the production of inflammatory substances such as C-reactive protein and interleukin 6 (IL-6) [36]. Psychological stress in pregnant women increases the production of adrenocorticotropic hormone-releasing hormone through the hypothalamic‒pituitary‒adrenal axis and stimulates the production of inflammatory cytokines [37]. PM2.5 also causes inflammation in the lungs and increases the production of interleukin 1β (IL-1β) [8].

Recently, it has been reported that being pregnant with a male fetus may suppress the maternal immune response [38, 39]. On the other hand, in women who are pregnant with a male fetus, nitrogen oxide is highly available and contributes to the response to vascular disorders, but it is thought that when the activity of reactive oxygen species is high, nitration damage and inflammation are promoted [40]. These reports suggest that being pregnant with a male fetus may suppress the maternal immune system because male fetuses are vulnerable to maternal inflammation, and if inflammation is promoted in the very early stage of pregnancy, it becomes difficult to maintain a pregnancy with a male fetus (early miscarriage).

It has been reported that pregnant women with high levels of cortisol, known as the stress hormone, have a higher risk of giving birth prematurely and giving birth to low-birth-weight babies, suggesting that the cortisol level may be a better measure than perceived stress for predicting birth outcomes [41, 42]. Elevated cortisol is also a known risk factor for spontaneous miscarriage [43], and it has been reported that exposure to air pollutants during early pregnancy may suppress fetal growth through an increase in cortisol [44]. Regarding the sex ratio at birth, women with higher levels of salivary cortisol are more likely to have girls [45].

In this study, based on the hypothesis that maternal inflammation just before and after fertilization or implantation increases the probability of failure to maintain pregnancy with a male fetus, this study investigated the correlation between the level of air pollutants at the time of fertilization or implantation and the sex ratio at birth. The decrease in the sex ratio at birth due to oxidant exposure, which was also observed in this study, may have been due to the fragility of male fetuses, making it easier to conceive female fetuses and resulting in a decrease in the sex ratio at birth.

### Limitations

Previous studies have reported that both paternal and maternal exposure to substances are factors that lower the birth sex ratio. Because this study only evaluated the relationship between atmospheric pollutant concentrations and the birth sex ratio, it was not possible to clarify whether this was due to paternal or maternal effects. In addition, various factors, such as maternal age, socioeconomic factors, medical history, and lifestyle habits during pregnancy, affect pregnancy and fetal development. Since this study used only data compiled and published within the jurisdiction of public health centers for analysis, it is necessary to clarify the influence of these individual maternal attributes and biomarkers, such as changes in female hormone and inflammatory cytokine levels, on the sex ratio at birth through more detailed research. Only monthly observation data were available for analysis. We could not calculate the individual timing of fertilization based on gestational age; thus, the relationship between daily air pollutant observational data and child sex could not be accurately evaluated. Further research is needed to clarify whether oxidant exposure has a strong effect on paternal gametes, ovaries, or the intrauterine environment.

## Conclusion

In this study, we considered the characteristics of seasonal variations in air pollutant levels on Fukue Island. We also evaluated the relationship between the observed concentrations of air pollutants and the sex ratio at birth and clarified that an increase in oxidant levels at conception was a risk factor for a lower sex ratio at birth. In this study, every 1 ppm increase in the observed OX value resulted in a 0.311 decrease in the sex ratio at birth in the model equation. Since it has been reported that events that decrease the sex ratio at birth are also risk factors for preterm birth, it is necessary to evaluate the effects of exposure to oxidants on pregnancy and childbirth from various aspects.

### Electronic supplementary material

Below is the link to the electronic supplementary material.

Supplementary Material 1

Supplementary Material 2

Supplementary Material 3

## Data Availability

The datasets used and/or analyzed during the current study are available from the corresponding author upon reasonable request.

## List of abbreviations

ANOVA: Analysis of Variance
IL-1β: Interleukin 1β
IL-6: Interleukin 6
MIC: Maximal Information Coefficient
CH_4_: Methane
NOx: Nitrogen Oxide
NMHCs: Nonmethane hydrocarbons
OXs: Oxidants
PM2.5: Particulate Matter 2.5
PM10: Particulate Matter 10
PCBs: Polychlorinated Biphenyls
SO_2_: Sulfur dioxide
VOCs: Volatile Organic Compounds
