# Supplementary figures and images for: Seasonal variation in air pollutant levels and its effects on the sex ratio at birth on Fukue island, Japan
Source: BMC Public Health. 2023 Dec 11;23:2471. doi: 10.1186/s12889-023-17418-5 (PMC10714618; doi:10.1186/s12889-023-17418-5)

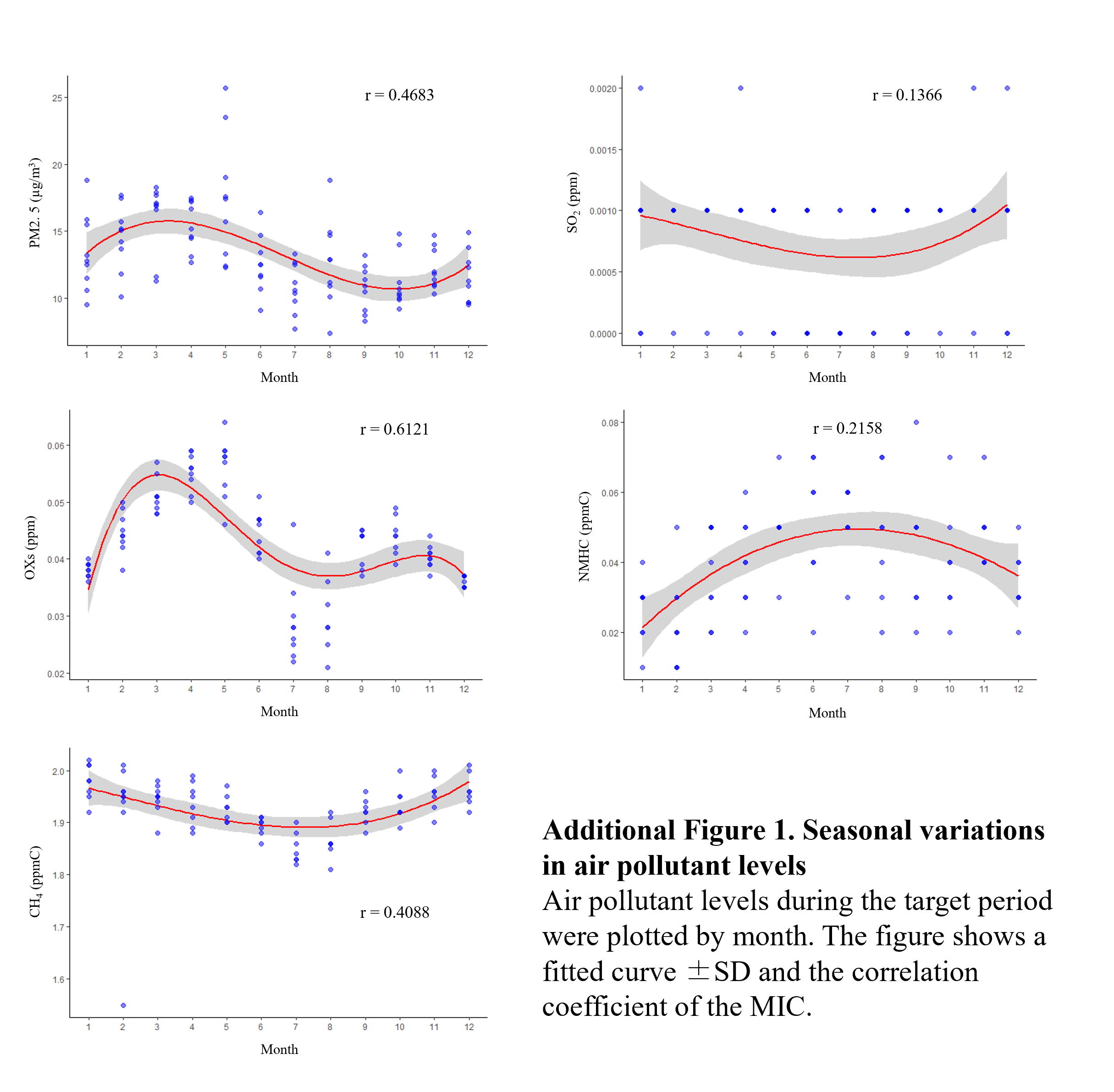

Supplement: Supplementary file 1 — Supplementary Material 1 [file 12889_2023_17418_MOESM1_ESM.png]

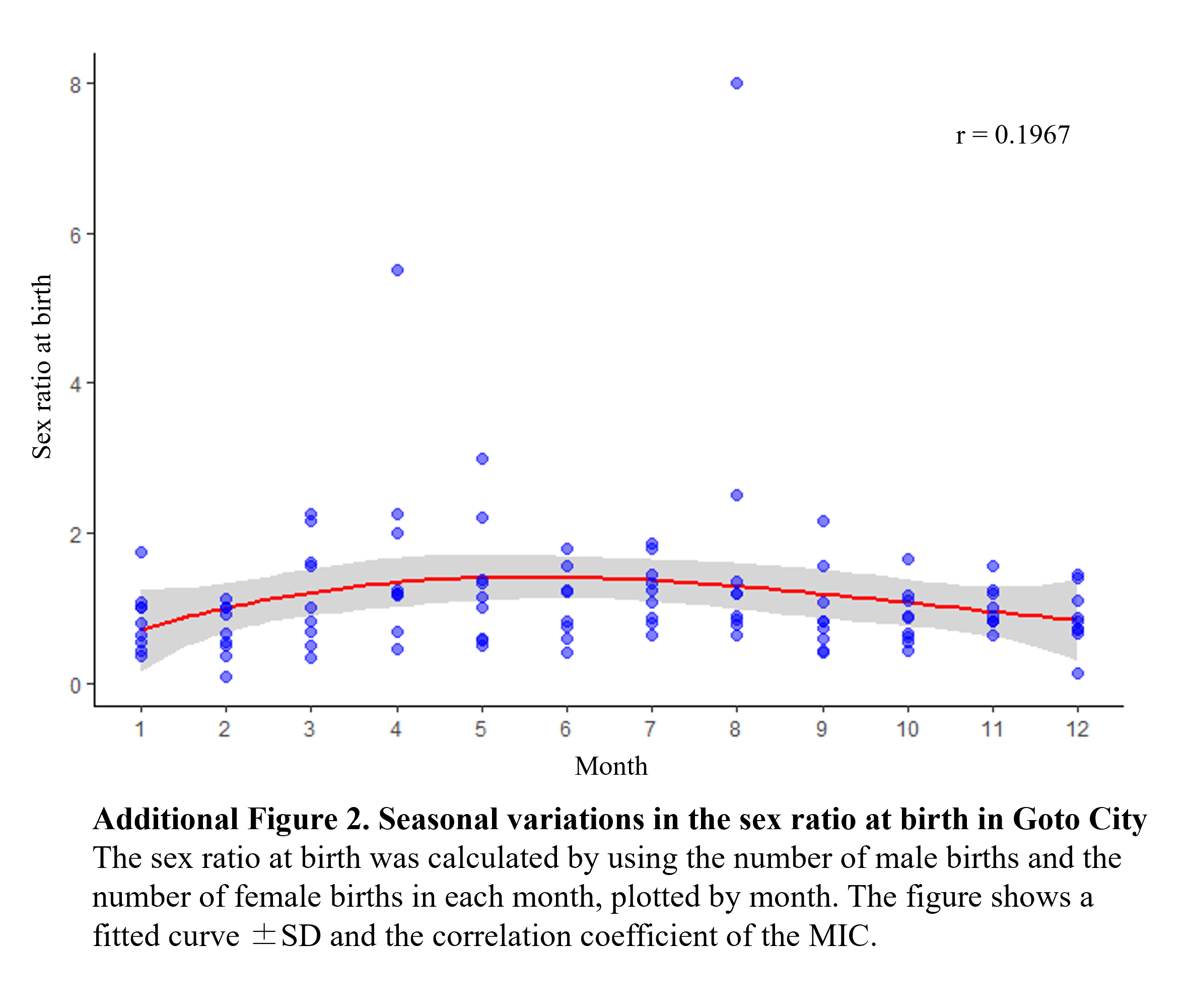

Supplement: Supplementary file 2 — Supplementary Material 2 [file 12889_2023_17418_MOESM2_ESM.png]
